# Computer-Aided Diagnosis of Acute Lymphoblastic Leukaemia

**DOI:** 10.1155/2018/6125289

**Published:** 2018-02-28

**Authors:** Sarmad Shafique, Samabia Tehsin

**Affiliations:** Department of Computer Science, Bahria University, Islamabad, Pakistan

## Abstract

Leukaemia is a form of blood cancer which affects the white blood cells and damages the bone marrow. Usually complete blood count (CBC) and bone marrow aspiration are used to diagnose the acute lymphoblastic leukaemia. It can be a fatal disease if not diagnosed at the earlier stage. In practice, manual microscopic evaluation of stained sample slide is used for diagnosis of leukaemia. But manual diagnostic methods are time-consuming, less accurate, and prone to errors due to various human factors like stress, fatigue, and so forth. Therefore, different automated systems have been proposed to wrestle the glitches in the manual diagnostic methods. In recent past, some computer-aided leukaemia diagnosis methods are presented. These automated systems are fast, reliable, and accurate as compared to manual diagnosis methods. This paper presents review of computer-aided diagnosis systems regarding their methodologies that include enhancement, segmentation, feature extraction, classification, and accuracy.

## 1. Introduction

Leukaemia is a cancerous growth of abnormal white cells which damages the blood and bone marrow. This rapid production of immature white blood cells (lymphoblast) disturbs the immune system of the body and reduces the bone marrow's ability to produce red blood cells and platelets [1]. Moreover, these abnormal leukaemia cells generally spread into the blood rapidly and can also invade other body parts like spleen, liver, kidney, lymph nodes, and brain, which may lead to other forms of cancer.

Diagnosis of leukaemia usually depends on the complete blood count (CBC) in which doctors check the complete count of white blood cells, red blood cells, and platelets. This complete blood count test may show leukaemia cells, but, in most cases, it is not enough for doctors to confirm that the patient has leukaemia. So, they have to use different methods including bone marrow aspiration [2] and microscopic examining of blood smear [3]. But all these manual methods require a lot of effort and time. Also, highly trained medical professionals are required to perform these types of examining and hence it is labor-intensive task. On the contrary, automated diagnostic systems can overthrow these problems of manual diagnosis. Furthermore, it will reduce the burden of medical professional and will provide accurate and effective results as compared to manual diagnosing.

Leukaemia is classified as either lymphocytic or myelogenous depending on which white blood cell (WBC) type is affected. If the immature cells are granulocytes and monocytes, then the leukaemia will be classified as myelogenous. If the immature cells are lymphocytes, then the leukaemia is classified as lymphocytic [4]. According to French American British (FAB) classification, acute lymphoblastic leukaemia is further categorized into 3 subtypes: L1, L2, and L3. These subtypes have different properties; for example, L1 type cells are usually small in size and are of similar shape with little cytoplasm. Their nucleus is circular and well structured. L2 type cells are different in shape and are usually larger than the L1 type cells. Their nucleus is irregular and they have variations in their cytoplasm. L3 type cells are of same shape and normal size with circular or oval shape nucleus. They have fair amount of cytoplasm which contains vacuoles. They are usually larger than L1.

Despite advancement, microscopic examination of blood smear still remains standard and hence economical method for leukaemia diagnosis. But this method of manual diagnosis depends on the operator, that is, his experience, exhaustion, personal issues, and so forth. Hence, these factors will significantly affect the outcome. So, there should be some effective and vigorous automated system for screening of leukaemia through which output results can be considerably improved without effect of operator's intervention. Furthermore, automated systems as compared to manual diagnosis can increase the accuracy and the speed of diagnosing. This will help the doctors to treat the leukaemia in more efficient manners. These methods can also play a vital rule in rural and underprivileged areas, where medical experts are not available.

This paper presents a detailed review of computer-aided acute lymphoblastic leukaemia (ALL) diagnosis methodology. This research also analyses and compares different methods over a range of parameters. This paper is organized as follows.  Section 2 describes materials and methods in the current literature including preprocessing techniques, segmentation methods, feature extraction, and classification methods.  Section 3 provides the discussion and analysis of work and Section 4 concludes the research.

## 2. Materials and Methods

Digital histopathology has witnessed a lot of improvement in the recent years. With the new technological advancement, much effective methods have been proposed for automated microscopic image analysis. Due to this development, computer-aided detection (CAD) is becoming a reliable method for ALL detection.

CAD system for ALL detection can be divided into four phases, namely, preprocessing, segmentation, feature extraction, and classification. This section provides a detailed survey of different techniques and methods that have been proposed, developed, and used in the automatic detection of acute lymphocytic leukaemia cell.

### 2.1. Preprocessing

In preprocessing step, image is enhanced and its quality is improved so that it can be accurately segmented and classified. There are lot of factors that adversely affect the quality of images, that is, false background, salt or pepper noise, and low contrast. These factors may occur due to the mishandling of camera, poor lightening conditions, and so forth. Different techniques are proposed by the researcher to detect these factors and to make the blood images suitable for the segmentation [5]. Histogram equalization is a very common method that utilizes the image histogram to adjust the contrast of image. This method is suitable for enhancing dark background/foreground and low contrast blood smear images [6]. Another very common technique is linear contrast stretching in which the intensity range of an image is extended to enhance the image. It is also called normalization of image. Many researchers have used this technique to enhance the quality of microscopic blood images by increasing the contrast [7, 8]. Selective median filtering with unsharp masking technique has been presented by Patil and Raskar which removes the noise from the images but maintains the edges, which helps to better identify the leukocytes and lymphocytes during the segmentation [9]. Many studies use minimum filter, which is used to highlight the lighter object to darker shade so that they can be easily recognized during segmentation process [10]. Another filter called order statistical filter is also used for the preprocessing of microscopic blood images, which removes the exponential noise as well as salt and pepper noise [11]. Gaussian filter with standard deviation is also applied, which reduces the noise and unwanted detail from the blood image to improve the accuracy for the efficient ALL identification [12]. Advantages and disadvantages of these methods are discussed in Table 1.

### 2.2. Lymphocyte Segmentation

**Figure 1:**
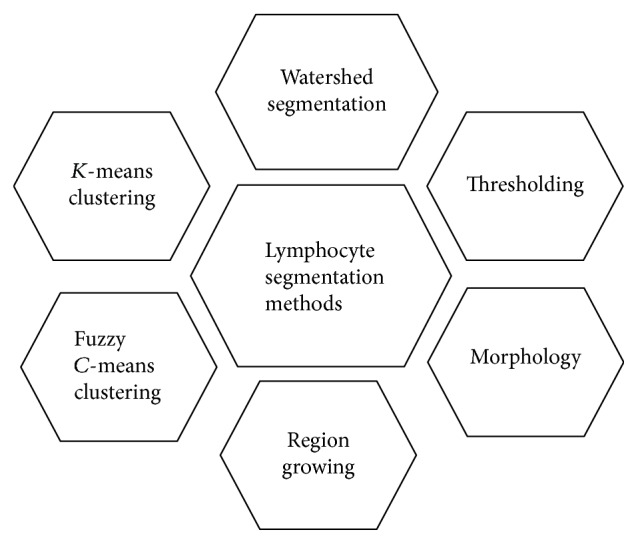
Lymphocyte segmentation methods.

Segmentation is the process of segregating the image into different regions through which we can easily understand the different parts of that image and we can analyse our region of interest from the segmented image [33]. Different pixels of the segmented image can have different labels which distinguish them from each other and the pixels having the same labels can share the characteristics of the pixels. Image segmentation is the significant part of acute lymphoblastic leukaemia detection, because based on precise segmentation a classifier will be able to classify the normal and blast cells accurately [34]. Plenty of image segmentation techniques have been presented in the literature to segment out white blood cells and lymphocytes to detect blast cells. Different methods for lymphocytes segmentation have been depicted in Figure 1.

#### 2.2.1. *K*-Means Clustering

*K*-means clustering is a semisupervised learning technique that is used when your data is not labeled. This algorithm is used to divide the image into *K* clusters by using *n* observations so that the objects in the same cluster are as close as possible and objects in the different cluster are different from other cluster's objects. This method has been used in the leukaemia blood image segmentation to extract the WBCs and lymphocytes from the image [35, 36].

Assume an initial set of *n* elements is given, and **m**_1_^(1)^,…, **m**_**k**_^(1)^ are *K*-means at time stamp 1; then the algorithm will be processed by alternating among the following 2 steps.

*1st Step: Assignment*. Accurately assign each observation to the nearest mean cluster.

Mathematically it can be defined as

(1) Sit=xp:xp−mit2≤xp−mjt2  ∀j,  1≤j≤k,

where each of the observations *x*_*p*_ will be assigned to one *S*^(*t*)^. 

*2nd Step: Update*. Now for the centroid of the observation calculate the new mean in the new cluster.

(2) $$\begin{array}{c} m_{i}^{\left(t+1\right)}=\frac{1}{\left|S_{i}^{\left(t\right)}\right|}\sum_{x_{j}\in S_{i}^{\left(t\right)}}x_{j}. \end{array}$$

This loop will continue till there is no/minimal difference in mean between two consecutive iterations.

#### 2.2.2. Fuzzy *C*-Means Clustering

Fuzzy *C*-means is the generalized form of *K*-means clustering. Unlike *K*-means clustering where each data point has its separate cluster, fuzzy *C*-means allow each data point to have same cluster. Fuzzy *C*-means is widely used in medical image segmentation because medical images have some signs of noise which is difficult for the hard clustering to tackle because in hard clusters like *K*-means clustering we have to define final number of *k* clusters; therefore they are sensitive to outliers [37]. On the other hand, fuzzy *C*-means takes more time as compared to *K*-means because of its fuzzy calculation measures. In ALL detection, this soft clustering algorithm plays a vital role because most of the leukaemia blood images have noise and sign of illumination, so fuzzy *C*-means gives better result, which helps in better classification of the disease but may take more time than *K*-means [26]. The basic fuzzy *C*-means algorithm is as follows:

1. Pick the desired number of clusters.
2. Randomly assign each data point to the cluster.
3. Repeat steps (1) and (2) until no more data points are left.

Fuzzy *C*-means algorithm divides *n* number of elements **A** = {**a**_1_, **a**_2_,…, **a**_**n**_} into fuzzy *c*-clusters **C** = {**c**_1_, **c**_2_,…, **c**_**n**_} and a partition matrix **M** = *m*_*ij*_ ∈ [0,1],  *i* = 1,…, *n*, *j* = 1,…, *c*, where every element *m*_*ij*_ tells to what degree element **a**_**i**_ belongs to cluster **c**_**j**_ with respect to some given criteria.

Mathematically it can be defined as

(3) $$\begin{array}{c} \underset{c}{\arg}\;min\overset{n}{\underset{i=1}{\sum }}\overset{c}{\underset{j=1}{\sum }}m_{ij}^{x}{\left\|\;a_{i}-\left.\;c_{j}\;\right\|\;\right.}^{2}, \end{array}$$

where

(4) $$\begin{array}{c} m_{ij}=\frac{1}{\sum_{k=1}^{c}{\left(\left\|a_{i}-c_{j}\right\|/\left\|a_{i}-c_{k}\right\|\right)}^{2/\left(x-1\right)}}. \end{array}$$

#### 2.2.3. Watershed Segmentation

Watershed is an image segmentation method that usually starts from the initial pixel called marker and uniformly deluges all the other neighboring pixels of that marker known as catchment basin. These basins are separated by the watershed, which will partition them into different regions or pixels. It is also categorized as region based segmentation technique [38]. Watershed segmentation is used in medical image processing because most of the WBCs in microscopic images are overlapping with each other; therefore they need to be separated in order to get better classification results. The main advantage of this method over other methods is that the regions that form from the resulting boundaries are closed and connected as compared to traditional edge based techniques that produce the boundaries which are disconnected and often need postprocessing to form closed regions [39]. Although watershed segmentation is a very simple technique and is suitable for parallel processing, due to existence of local minima, it can produce oversegmentation. So, for acute lymphoblastic leukaemia detection, most of the studies have used marker controlled watershed segmentation to segment out the leukaemia cells [40], which use predefined internal and external markers. In marker controlled watershed boundaries can be predefined or they can be defined as ridges between external and internal markers. External marker in this method is manually defined according to our object of interest and internal marker is automatically obtained by the combination of different techniques like thresholding, edge detection, and morphological operations. This method will tackle the oversegmentation problem and help in segmenting the objects with closed form [41]. Mathematically it can be written as

(5) $$\begin{array}{c} LS\left(\;x\;\right)=\underset{y\in N\left(x\right)}{max}\left(\;\frac{I\left(x\right)-I\left(y\right)}{d\left(x,y\right)}\;\right), \end{array}$$

where LS(*x*) is the lower slope of image *I* at pixel *x* and *N*(*x*) is the neighboring pixel set of the pixel *x* and *d*(*x*, *y*) is the watershed distance between *x* and *y*. If *i* = *j*, the LS (lower slope) will be assigned zero. The cost from pixel *x* to *y* can be calculated as

(6) costx,y=LSx·dx,yif  fx>fyLSy·dx,yif  fx<fy12LSx+LSy·dx,yif  fx=fy.

#### 2.2.4. Thresholding

Thresholding is another effective image segmentation method that is used to convert the gray scale image into binary image. By partitioning the image into background and foreground, we can easily eliminate its background to get our desired objects. If an image has high contrast, then the thresholding will be very effective for its segmentation [42]. This method works with a standard threshold value; if the intensity value of the image is less than the threshold value, then the value will be zero, that is, convert to black color, and if the value of the intensity level is greater than the threshold value, then the intensity value will be converted to white. Thus, this will generate a binary image with separated foreground and background, which will help in better understanding of the image [43]. Mathematically, it can be written as

(7) $$\begin{array}{c} I^{\prime }\left(\;i\;\right)=\left\{\begin{array}{cc} 1, & \text{if }I\left(i\right)\geq T\\ 0, & \text{otherwise,} \end{array}\right. \end{array}$$

where *I* is the original grey scale image with *i*th pixel value in the image. *I*′ is the binary image if *T* is a global threshold value.

In medical image segmentation, thresholding is an effective method for image segmentation. For acute lymphoblastic leukaemia detection, one of the most used thresholding techniques is Otsu's method, which finds the optimal threshold value by the minimal variance in the class to separate the lymphocytes from the blood leukaemia image [44]. Although Otsu's thresholding gives good results, being a global thresholding technique it can blur the local edges of blood cells, so to avoid this problem different edge-preserving filters are used to preserve sharp edges of blood cells. Another triangle oriented thresholding method has been used for lymphocytes detection by using Zack's algorithm. This algorithm constructs a line between highest histogram value of the image and lowest histogram value through which an optimal threshold value is calculated and image is segmented based on that threshold value [45]. For acute lymphoblastic leukaemia detection, Otsu's thresholding [16, 21] method performs better and provides 93% overall accuracy as compared to Zack's algorithm [17, 29] that is able to achieve 92% results.

#### 2.2.5. Region Growing

It is a region based segmentation technique that is used to select the region of interest from the image by using predefined conditions. By utilizing edge detail of an image, a condition can be defined for region of interest selection [46]. It is also called pixel based segmentation because an initial pixel is selected in the image and then its neighboring pixels that are connected with the initial pixel having the same intensity value are selected as a region of interest. However, selecting the pixels manually is not a proficient approach. But still it is widely used in the medical image segmentation for picking tumor cells from the microscopic image [47].

Region growing is basically used to divide the image into different regions. Its basic formula is as follows:

- ⋃_*i*=1_^*n*^*R*_*i*_ = *R*.
- *R*_*i*_ is a connected region, *i* = 1,2, 3,…, *n*.
- *R*_*i*_∩*R*_*j*_ = *ϕ* for all *i* = 1,2,…, *n*.
- *P*(*R*_*i*_) =* TRUE* for *i* = 1,2,…, *n*.
- *P*(*R*_*i*_ ∪ *R*_*j*_) =* FALSE* for any adjacent region *R*_*i*_ and *R*_*j*_.

(a) shows that every pixel should be the part of the same region. (b) indicates that all pixels in the region should be connected; (c) shows that all the regions must be disjoint from each other. (d) shows properties that should be true or false with respect to some criteria. (e) indicates that the regions should be different with respect to predicate *P*.

#### 2.2.6. Morphology

Morphology or mathematical morphology is a set theory based technique that is used to extract different components from the image for the better representation and description for the shapes and regions [48]. Morphological image processing deals with the shape of different objects in an image by using some specific image processing techniques. The basic idea behind the morphology is to slide a predefined small shape called structuring element over the image *I* so that we can get our desired result depending on whether the structuring elements fit or miss over the image [49].

There are two basic morphological operators, which are erosion ⊖ and dilation ⊕. Let *E* be a binary image and let *S* be a structuring element. Then the erosion ⊖ and dilation ⊕ of the binary image *E* with the structuring element *S* can be defined as

(8) E⊖S=z ∣ Sz⊆E,E⊕S=z ∣ S^z∩E≠ϕ.

Erosion is basically used to shrink the foreground objects and smooth their boundaries. Dilation expands the foreground objects and smoothens their boundaries and fills the small holes and gaps. There are two more morphological operations called opening ∘ and closing •.

The opening of an image *E* by the structuring element *S* is an erosion followed by dilation.

(9) $$\begin{array}{c} E\circ S=\left(\;E⊖S\;\right)⊕S. \end{array}$$

Closing of an image *E* by structuring element *S* can be defined as dilation followed by erosion.

(10) $$\begin{array}{c} E•S=\left(\;E⊕S\;\right)⊖S. \end{array}$$

Top-hat transform is another morphological operation that is divided into two types called white top-hat transform and black top-hat transform. These operators are used to extract small image details and element information from the given image.

Black-hat transform is the difference between image *I* and its closing, which is defined as

(11) $$\begin{array}{c} T_{b}\left(\;I\;\right)=E-\left[\;E•S\;\right]. \end{array}$$

White-hat transform is defined as the difference between an image *I* and its opening, which is defined as follows:

(12) $$\begin{array}{c} T_{w}\left(\;I\;\right)=E-\left[\;E\circ S\;\right]. \end{array}$$

In medical image segmentation, morphology plays an important role. In ALL detection, many researchers have used morphological operation for the segmentation of leukaemia cells. It efficiently enhances the cells by filling small holes and gaps, smoothening their boundaries, and removing the salt or pepper noise from the nucleus. It is usually used with watershed transform to segment out the blast cells and to identify the connected cells for the separation of blast cells [50].

### 2.3. Features Extraction

In digital image processing and machine learning, features are the information we retrieve from the computational problem, which helps us to efficiently solve the task. Features can be some specific structure of the image like its shape, points, texture, edges, and so forth. [51]. Features extraction is the interpretation of this information to reduce the dimension of image in such a way that is more informative and less redundant. This technique is very effective when the algorithm has a large set of data and that data can be also redundant; then the data is minimized to reduce set of features that carry the most of information of the image and is easy to compute by the algorithm. The selection of subset of these relevant features is called features selection [52]. These features will contain the most relevant information and this subset is used instead of complete feature set. By minimizing the information into reduced set of features, a classifier will give better result by interpreting fewer amounts of data with more relevant information.

**Figure 2:**
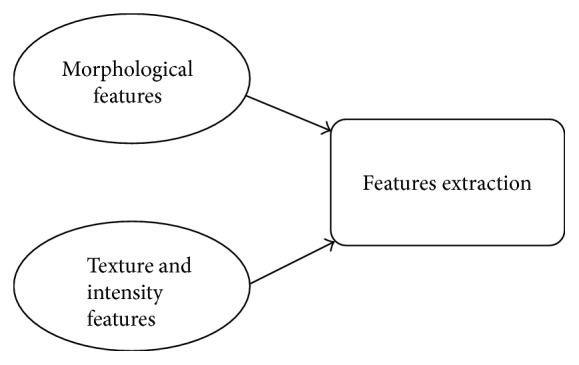
Features extraction techniques.

For acute lymphoblastic leukaemia detection, features extraction plays vital role because blast cells may have lot of information including different characteristics of their nucleus and cytoplasm [53]. Different features have been extracted in the recent study. These features can be divided into two broad categories, morphological features and texture features, as shown in Figure 2.

#### 2.3.1. Morphological Features

In medical image processing, morphological features are very effective to analyse the information of the blood cell. In acute lymphoblastic leukaemia, blast cell has unique shape based features because every type of cell has unique area, perimeter, and circular rounding. So, by extracting morphological features from the blood cells we can efficiently perform classification of these cells [54]. 

*(1) Shape Features*. Shape based features play very important role for the acute leukaemia cell detection. Different shape based features like area, perimeter, circulatory, solidity, eccentricity, and so forth have been extracted to classify the blast cells of leukaemia [55]. 

*(2) Bending Energy*. Bending energy is also an essential feature that helps in efficient detection of acute lymphoblastic leukaemia. This parameter is used to detect the curvature of blast's cell boundary which can help in ALL classification [56]. 

*(3) Roundness Ratio*. Roundness ratio is also an important feature that is widely used in leukaemia detection. Because of increased variance in the circular shape of blast cells, roundness ratio is an efficient feature for the better classification of leukaemia cell and its subtypes. Also, it helps in counting of WBCs which is also an important factor for the leukaemia occurrence [57]. 

*(4) Chain Code*. Chain code features are widely used in acute lymphoblastic leukaemia detection. These features separate the boundaries of nucleus and cytoplasm of blast cell which will help us to trace out the nucleus and cytoplasm [58].

#### 2.3.2. Texture and Intensity Features

In medical image processing, texture is an important characteristic for the identification of blast cells. By analysing texture of an image, we can easily pick our region of interest and it also describes the spatial intensity distribution and specific colors in that ROI. For leukaemia detection, important information including texture and intensity has been extracted from blood smear images, which can help in better classification of blast cells for leukaemia detection and blast cell identification.

*(1) GLCM (Grey Level Cooccurrence Matrix)*. GLCM is a statistical method used for the examining of texture in which spatial relationship between the pixels is considered. For leukaemia detection, GLCM is very useful to utilize the texture of the input blood smear image and extract features based on the texture and intensity of blast cells [59]. 

*(2) Histogram*. Histogram features including entropy, energy, mean, standard deviation, skewness, and kurtosis are extracted from the blood smear image to get enough relevant information. These types of features are also called 1st-order statistical features that are calculated by utilizing original pixels and excluding neighbour pixels [18]. 

*(3) Gabor Texture Extraction*. Gabor texture extraction method proposed by Dennis Gabor is very useful to extract relevant information of a blood smear image by analysing its texture. Gabor features can be extracted after applying Gabor filter [60]. 

*(4) Color Intensity Features*. Color features are very useful for fetching relevant information from blood cell nucleus. So, mean color values from different color models like RGB, HSV, HIS, and so forth are extracted as a feature and input to the classifier for better classification of blast cells [61]. 

*(5) Fractal Dimension Features*. Fractal dimension is widely used in medical image processing to measure different quantitative information. To identify whether a leukaemia cell is blast or normal, the roughness of its nucleus is being measured over spatial distribution by using fractal geometry [62]. 

*(6) Entropy*. By performing nucleus texture measurement, we can extract the entropy as a feature vector that is used to measure the randomness of the nucleus from the blood smear image. Different entropy measurements are used for the acute lymphoblastic leukaemia cell detection [63]. 

*(7) Hausdorff Dimension*. It is also an essential feature for microscopic blood image analysis, which is used with fractal dimension to extract relevant information by measuring roughness of nucleus [64]. 

*(8) Local Binary Pattern*. Local Binary Pattern is a texture classification technique that is used to extract texture features of an image. Because of fast computational speed of LBP, it is highly recommended for leukaemia detection, where speed is an important factor [65, 66]. Also, it provides significant information about the illumination changes, which also helps in detection of blasted leukaemia cells. In [67], Discriminative Robust Local Binary Pattern is proposed for features extraction, which provides very encouraging results.

#### 2.3.3. Features Selection

Features selection is a technique used in pattern recognition and computer vision, which helps in selecting those important features that are more relevant to the problem and are not redundant. This will allow the algorithm to select a subset of features, which will increase the efficiency and accuracy and will reduce the computational cost due to reduced input [68]. Therefore, feature selection will compare all the initial features that are extracted from the microscopic blood smear images and select the most relevant features. For acute lymphoblastic leukaemia detection, different feature selection techniques are used. In [69], PCA (Principal Component Analysis) technique is used to reduce the features to avoid any redundancy. Genetic Algorithm is also used to select important features [70, 71]. PPCA (Probabilistic Principal Component Analysis) technique also gives better performance for features reduction [13].

### 2.4. Classification Methods for ALL Detection

**Figure 3:**
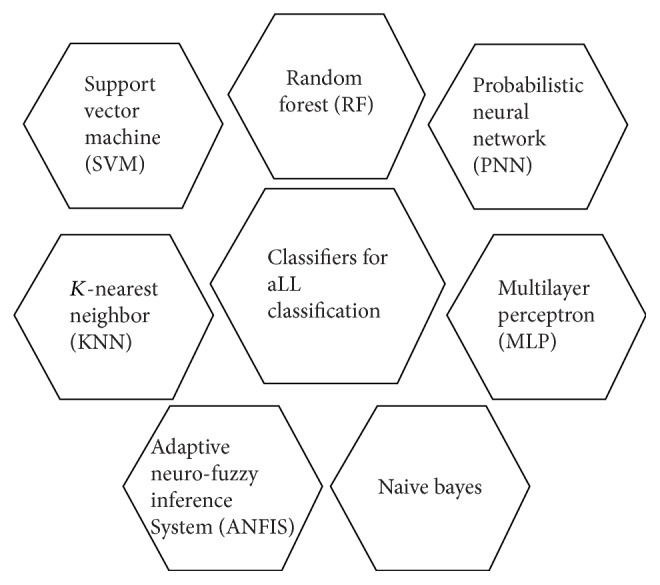
Classification methods for ALL.

Computer-aided diagnosing is an effective method to accurately diagnose acute lymphoblastic leukaemia. Computer-aided method can be fully automated or semiautomated depending on the approach you are using. ALL (acute lymphoblastic leukaemia) is a pattern classification problem that is diagnosed by extracted features of microscopic blood smear images. Classification is a supervised learning technique that uses training data to train its model and test data to check performance of that model [72]. Different pattern classifiers are used for diagnosis of ALL, which are summarized in Figure 3.

#### 2.4.1. SVM (Support Vector Machine)

Support vector machine is one of extensively used algorithms for leukaemia detection. This algorithm is used to output and optimize hyperplane that classifies the given data based on their features [21, 73]. The main reason behind the selection of SVM for leukaemia detection is that it is a binary classifier that can efficiently classify between normal and blast cells. However, a custom approach has been used for classifying its subtypes like L1, L2, and L3 [74].

#### 2.4.2. KNN (*K*-Nearest Neighbor)

*K*-nearest neighbor is a widely used classification and regression technique that uses the nonparametric and lazy learning method to classify different data. In *k*-nearest neighbour algorithm, classification is done by the voting from the nearest neighbours. Based on this voting, objects will be assigned to their relevant classes. For acute lymphoblastic leukaemia cell classification, *k*-NN classifier is used to get better classification result for the normal and blast cells [75].

#### 2.4.3. Random Forest

Random forest is an efficient classification technique that uses ensemble learning method to classify an object from the input vector. It contains different combinations of trees which perform voting for the selection of the class and then selection will be based on the class having maximum vote [76]. Although random forest depends on random numbers due to which it may give different results every time, still this classifier is preferred by the researcher because it corrects the problem of overfitting. For acute lymphoblastic leukaemia detection, random forest has been found to be stronger classifier for the detection of normal and infected blood cells [77].

#### 2.4.4. ANFIS (Adaptive Neurofuzzy Inference System)

Adaptive neurofuzzy inference system is another good classifier that is widely used for the medical image classification. This is a very powerful method because it is the combination of artificial neural network and fuzzy logic. Although its design complexity is more than other classifiers like random forest, ANFIS is an end-to-end classifier as compared to random forest performs classification using given features, so if features are not strong enough random forest cannot performs classification accurately. Therefore ANFIS helps in better classification and early diagnosis of many diseases [78]. Recent study shows that ANFIS has been used for the classification of blast cell in leukaemia patients by microscopic blood image analysis, where it gives appropriate results [79].

#### 2.4.5. Naive Bayes

Naive Bayes is an efficient classifier that is used to categorize the data by applying Bayes' theorem. In this classifier, values of features are assumed to be independent of other feature values [80]. Naive Bayes has multiple names like simple Bayes and independent Bayes. For acute lymphoblastic leukaemia detection, Naive Bayes has been utilized in the recent study, where it is used to efficiently classify the normal and blast cell from the microscopic blood images [81].

#### 2.4.6. Multilayer Perceptron (MLP)

Multilayer perceptron is an artificial neural network model that contains multiple layers of node and each layer is connected to its next node. In MLP, each input node represents input data and all other nodes are neuron that gives output by using activation function. It is widely used in large-scale problems because of its simple design and powerful computational capability. Recent study shows that MLP has been utilized for the acute lymphoblastic leukaemia detection, where it uses scaled conjugate gradient back propagation (SCG) for training blood images and on basis of this trained model performs classification to classify normal and cancerous cells [61].

#### 2.4.7. Probabilistic Neural Network (PNN)

Probabilistic neural network is a widely used classifier. In PNN classifier, Parzen window as well as a nonparametric function is used to approximate probability distribution function of each class, through which probability of input data can be estimated and by applying Bayes rule each class with highest probability will be assigned to the input data [82]. For acute lymphoblastic leukaemia detection, probabilistic neural network has been used for the efficient classification of normal and blast cells [83].

## 3. Analysis and Discussion

We have presented a basic overview of image analysis and machine learning techniques that are used for the acute lymphoblastic leukaemia detection and classification of normal and blast cells. We have briefly discussed all the methods and techniques that have been used in previous research to check how they were implemented and what are their pros and cons as compared to the manual diagnosing methods available for leukaemia detection. These computer-aided methods are found to be more efficient, reliable, accurate, and less time-consuming as compared to the manual diagnosing methods that were less efficient and more time-consuming.

Table 2 shows a systematic comparison of various ALL diagnostic methods. Every method has used its own technique to diagnose acute lymphoblastic leukaemia. Different preprocessing, segmentation, features extraction, and classification techniques with their performance have been shown in Table 2.

Karthikeyan and Poornima [14] used histogram equalization and median filtering for preprocessing of ALL images and then fuzzy *C*-means was used to segment out the white blood cells and after extracting features using Gabor texture extraction method support vector machine was used for classification of blasted cells. They were able to achieve 90% accuracy by using above method. Putzu and Ruberto [17] had improved their accuracy to 92% by using triangle threshold with improved features. They had extracted shape, color, and texture features using GLCM. After that, SVM was used for classification of blast and normal cells. Mohapatra et al. [27] achieved an accuracy of 94.73% by using their proposed methodology. They used contrast enhancement and selective median filtering for noise removal and contrast adjustment, which helps in better segmentation of leukaemia images. Shadowed *C*-means clustering was used for the segmentation of lymphocytes which clustered the lymphocytes into 3 regions including their background, cytoplasm, and nucleus. After that, different features including fractal dimension, shape based features, color features, and texture features were extracted from the segmented lymphocytes. Then a powerful ensemble classifier (Naive Bayesian, *K*-nearest neighbor, multilayer perceptron, radial basis functional neural network, and support vector machines) was used to classify normal and blast cells. Mishra et al. [13] used histogram equalization and Weiner filtering to enhance contrast and noise adjustment. Improved watershed segmentation with Sobel and Prewitt operators was used to achieve better segmentation. After extracting GLCM based texture features, they used random forest classifier rather than SVM, which improved the accuracy to 96.29% as compared to other methods. Savita Dumyan [20] increased their accuracy to 97.8% by using canny edge detection that is efficient and reliable as compared to other edge detection techniques because of its low error rate. After extracting features, powerful ANN (artificial neural network) classifier was trained to classify normal and blast cells. Li et al. [18] proposed novel segmentation technique for white blood cells, where dual-threshold method was used for segmentation, in which optimal threshold value was determined by using golden section search. After that, postprocessing was carried out, in which morphological operation and median filtering were used to enhance the segmentation results. By using this method, they were able to achieve accuracy of 98%. Rawat et al. [15] were able to achieve higher accuracy of 99% as compared to all previous methods by using powerful hybrid hierarchical classifiers. Histogram equalization and order statistic filter were used for contrast adjustment and noise removal. Global thresholding followed by morphological opening was used for segmentation of lymphocytes. After that different features were extracted and reduced by using PCA (Principal Component Analysis). Then 2-method classification technique was used, in which five classifiers were arranged in hierarchal order to detect normal and blast cells; then subtypes of acute lymphoblastic leukaemia, that is, L1, L2, and L3, were also classified by using this method.

**Figure 4:**
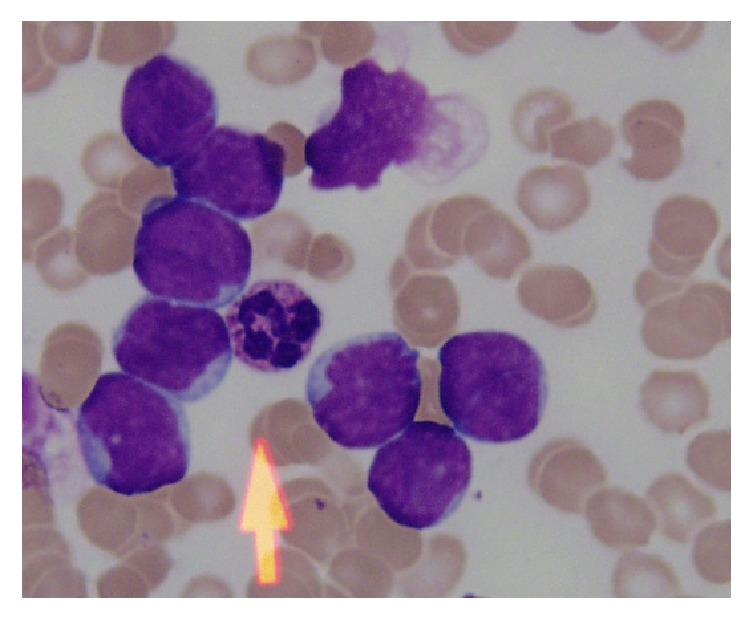
Overlapping cells.

After analysing the previous methods, we come to the point that there is still research room available to improve the accuracy of leukaemia detection. ALL detection and diagnosis are very sensitive issues and are related to the health and life of humans. Accuracy of such diagnosis process should be flawless for complete replacement of human operators by these computer-based diagnoses but this is a very challenging task because these blood cells are highly overlapping as shown in Figure 4, which makes them difficult to separate.

**Figure 5:**
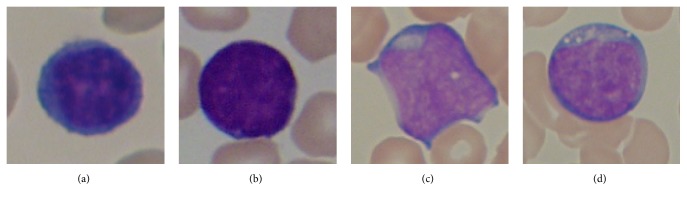
Subtypes of ALL according to FAB. (a) A noncancerous cell, (b) L1 type ALL, (c) L2 type ALL, and (d) L3 type ALL.

It is also analysed that classification of ALL subtypes is somewhat ignored in the literature. Most researchers have neglected the identification of subtypes of acute lymphoblastic leukaemia because of their interclass similarity and intraclass variability. These subtypes are difficult to classify but play vital role in precise diagnosis of disease and are very crucial for the medical treatment of the disease. Different subtypes of acute lymphoblastic leukaemia are shown in Figure 5. According to FAB classification, we have the following:

- *L1*: these blast cells are homogenous and small with very scanty cytoplasm and do not contain vacuoles. Their nucleus is regular and round
- *L2*: they are heterogeneous and large in size with irregular nucleus. Cytoplasm may contain vacuoles
- *L3*: these blast cells are homogenous and their size is moderate. Their nucleus is round and regular and cytoplasm has notable vacuoles.

## 4. Conclusion and Future Prospects

In this paper, we have provided the brief detail about ALL. We also highlighted different methods for diagnosis of acute lymphoblastic leukaemia (ALL) which have been proposed by different researchers. ALL is a fatal disease that needs to be diagnosed earlier for proper treatment. So, we have compared different methods for the early detection of leukaemia. Techniques for different stages of the diagnostic methods are summarized, analysed, and compared in this research. Although a lot of research work has been done for ALL detection, there is tremendous amount of work needed to make its detection flawless, accurate, cost-effective, and more efficient. Also, after comparing the previous work, we come to the point that there are very few works done for the classification of its subtypes, which have opened more room for researchers to work on subtypes of leukaemia, which will help the pathologist to do effective treatment of leukaemia patient.

## Figures and Tables

**Table 1:** Advantages and disadvantages of different preprocessing methods.

Method	Advantages	Disadvantages
Histogram equalization	Simple technique to enhance the contrast of an image by utilizing its histogram. Useful for images having darker or brighter background and foreground.	It is only useful if the input image has low contrast; otherwise it can reduce the image quality. It cannot discriminate between noise and actual image, which can increases the contrast of noise.

Linear contrast stretching	Enhance the contrast of image by extending dynamic range of intensity values. Useful for low contrast images.	Main disadvantage of linear contrast stretching is that it is vulnerable to noise. An image having single outlier pixel can reduce the effectiveness of this operation.

Median filter	Popular nonlinear filter for removing salt and pepper noise. Useful for preserving sharp edges of image.	Only useful for images having low density noise. It cannot perform well for the images having high percentage of salt and pepper noise.

Minimum filter	Easy to implement. Useful for removing salt noise from the images.	Can remove image detail if images are highly noisy.

Gaussian filter	Useful for removing blur and noise from the images.	If used alone can blur the edges and reduce the contrast of images.

Unsharp masking	Simple method for image sharpening. Remove blurriness from the image.	Highly sensitive to noise because of linear high pass filter.

**Table 2:** Comparison of different acute lymphoblastic leukaemia detection methods.

Authors, year	Method	Dataset	Preprocessing	Segmentation	Features extraction	Classification	Performance%
Mishra et al., 2017	Gray level cooccurrence matrix and random forest based acute lymphoblastic leukaemia detection [13]	Public ALL-IDB 1 (108 images)	Histogram equalization, Weiner filtering	Sobel, Perwitt, Marker based watershed segmentation	GLCM (gray level cooccurrence matrix), PPCA (Probabilistic Principal Component Analysis)	RF (random forest)	Accuracy 96.29%

Karthikeyan and Poornima, 2017	Microscopic image segmentation using fuzzy *C*-means for leukaemia diagnosis [14]	Google (19 images)	Histogram equalization, Median filter	*K*-means clustering, fuzzy *c*-means clustering algorithm	Gabor texture extraction method	SVM (support vector machine)	Accuracy 90%

Rawat et al., 2017	Classification of acute lymphoblastic leukaemia using hybrid hierarchical classifiers [15]	Public ALL-IDB 2 (260 images)	Histogram equalization, Order statistic filter	Global thresholding, morphological opening	Geometrical features, chromatic features, statistical texture features	Hybrid hierarchical classifiers kNN, PNN, SVM, SSVM, and ANFIS	Accuracy 99.2%

Joshi et al., 2013	White blood cells segmentation and classification to detect acute leukaemia [16]	Public ALL-IDB 1 (108 images)	Contrast Stretching and Histogram equalization	Otsu's threshold method	Shape features (area, perimeter, and circularity)	KNN (*K*-nearest neighbor)	Accuracy 93%

Putzu and Ruberto, 2013	White blood cells identification and classification from leukaemia blood image [17]	Public ALL-IDB 1 (108 images)	Histogram equalization	Triangle threshold method using Zack algorithm	Shape based features (area, perimeter, etc.), GLCM features	SVM (support vector machine)	Accuracy 92%

Li et al., 2016	Segmentation of white blood cell from acute lymphoblastic leukaemia images using dual-threshold method [18]	Public ALL-IDB (130 images)	Global Contrast Stretching	Dual-threshold segmentation	Binarization, morphological erosion, median filtering (postprocessing)	Not mentioned	Accuracy 98%

Amin et al., 2015	Recognition of acute lymphoblastic leukaemia cells in microscopic images using *K*-means clustering and support vector machine classifier [19]	Isfahan Al-Zahra and Omid Hospital pathology laboratories (146 ALL images and 166 lymphocytes images)	Histogram equalization	*K*-means clustering	Shape based features (area, perimeter, solidity, and eccentricity), histogram-based features (mean, standard deviation skewness, entropy, etc.)	SVM (support vector machine)	Accuracy 95.6%

Savita Dumyan, 2017	An enhanced technique for lymphoblastic cancer detection using artificial neural network [20]	Blood sample images (36 images)	Histogram equalization	Image binarization, canny edge detection technique	Shape based features, texture features, statistical features, moment invariants	Artificial neural network (ANN)	Accuracy 97.8%

Chatap and Shibu, 2014	Analysis of blood samples for counting leukaemia cells using support vector machine and nearest neighbour [21]	Public ALL-IDB 1 (108 images) and ALL-IDB 2 (260 images)	Histogram Equalization, Contrast Stretching	Otsu's threshold method	Shape based features (area, perimeter, and circularity)	*K*-nearest neighbor (KNN)	Accuracy 93%

Amin et al., 2015	Recognition of acute lymphoblastic leukaemia cells in microscopic images using *K*-means clustering and support vector machine classifier [22]	Isfahan Al-Zahra and Omid Hospital pathology laboratories (312 images)	Histogram Equalization, Linear Contrast Stretching	*K*-means clustering	Geometric or shape based (area, perimeter, convex, and solidity), first- and second-order statistical features	SVM (support vector machine)	Accuracy 97% (blast and normal), 95.6% (subtypes classification)

Patel and Mishra, 2015	Automated leukaemia detection using microscopic images [23]	Not mentioned	Median Filtering, Wiener Filtering	*K*-means clustering	Color features, geometric, texture, and statistical features	SVM (support vector machine)	Accuracy 93.57%

MoradiAmin et al., 2016	Computer aided detection and classification of acute lymphoblastic leukaemia cell subtypes based on microscopic image analysis [24]	Isfahan Al-Zahra and Omid Hospital pathology laboratories (312 images)	Histogram equalization	Fuzzy *C*-means, watershed algorithm	Geometric or shape based (area, perimeter, convex, and solidity), first- and second-order statistical features	SVM (support vector machine)	Accuracy 97.52%

Mohapatra and Patra, 2010	Automated cell nucleus segmentation and acute leukaemia detection in blood microscopic images [25]	University of Virginia, Ispat General Hospital, Rourkela, Odisha (108 images)	Selective median filtering, Unsharp Masking	*K*-means clustering, nearest neighbor	Fractal dimension, shape features including contour signature and texture, color features	SVM (support vector machine)	Accuracy 95%

Mohapatra et al., 2011	Fuzzy based blood image segmentation for automated leukaemia detection [26]	University of Virginia, Ispat General Hospital, Rourkela, Odisha (108 images)	Selective median filtering, Unsharp Masking	Gustafson Kessel clustering, nearest neighbor	Fractal dimension, shape features including contour signature and texture, color features	SVM (support vector machine)	Accuracy 93%

Mohapatra et al., 2014	An ensemble classifier system for early diagnosis of acute lymphoblastic leukaemia in blood microscopic images [27]	Ispat General Hospital, Rourkela, Odisha (150 images)	Contrast enhancement, Selective median filtering	Shadowed *C*-means (SCM) clustering	Fractal dimension, shape features including contour signature and texture, color features	Ensemble method (Naive Bayesian, *K*-nearest neighbor, multilayer perceptron, radial basis functional neural network, support vector machines)	Accuracy 94.73%

Samadzadehaghdam et al., 2015	Enhanced recognition of acute lymphoblastic leukaemia cells in microscopic images based on feature reduction using principle component analysis [28]	Isfahan Al-Zahra and Omid Hospital pathology laboratories (21 Images)	Histogram equalization	Fuzzy *C*-means, watershed algorithm	Geometric or shape based (area, perimeter, convex, and solidity), statistical features	SVM (support vector machine)	Accuracy 96.33%

Putzua et al., 2017	Leucocyte classification for leukaemia detection using image processing techniques [29]	Public ALL-IDB 1 (108 images) and ALL-IDB 2 (260 images)	Histogram equalization and contrast stretching	Zack algorithm	Shape features, color features, texture features	SVM (support vector machine)	Accuracy 92%

Sadeghian et al., 2009	A framework for white blood cell segmentation in microscopic blood images using digital image processing [30]	L2 type ALL blood images (20 images)	Gaussian filter, Standard deviation	Canny edge detection technique, Zack algorithm	Not mentioned	Not mentioned	Accuracy 92% (nucleus segmentation), 78% (cytoplasm segmentation)

Mohapatra and Patra, 2010	Automated leukaemia detection using Hausdorff dimension in blood microscopic images [31]	University of Virginia, Ispat General Hospital, Rourkela, Odisha (108 images)	Selective median filtering, Unsharp masking	*K*-means clustering	Hausdorff dimension, shape features, color features	SVM (support vector machine)	Accuracy 95%

Mohapatra et al., 2010	Image analysis of blood microscopic images for acute leukaemia detection [32]	University of Virginia, Ispat General Hospital, Rourkela, Odisha (108 images)	Selective median filtering, Unsharp masking	Fuzzy *C*-means clustering, nearest neighbor	Fractal features (Hausdorff dimension), shape features, contour signature, color, texture features	SVM (support vector machine)	Accuracy 95%
